# Clinical and socioeconomic predictors of hospital use and emergency department visits among children with medical complexity: A machine learning approach using administrative data

**DOI:** 10.1371/journal.pone.0312195

**Published:** 2024-10-29

**Authors:** Michael Sidra, Matthew Pietrosanu, Jennifer Zwicker, David Wyatt Johnson, Jeff Round, Arto Ohinmaa

**Affiliations:** 1 School of Public Health, University of Alberta, Edmonton, Canada; 2 Department of Mathematical and Statistical Sciences, University of Alberta, Edmonton, Canada; 3 Faculty of Kinesiology, School of Public Policy, University of Calgary, Calgary, Canada; 4 Department of Pediatrics, University of Calgary, Calgary, Canada; Chitwan Medical College, NEPAL

## Abstract

**Objectives:**

The primary objective of this study was to identify clinical and socioeconomic predictors of hospital and ED use among children with medical complexity within 1 and 5 years of an initial discharge between 2010 and 2013. A secondary objective was to estimate marginal associations between important predictors and resource use.

**Methods:**

This retrospective, population-cohort study of children with medical complexity in Alberta linked administrative health data with Canadian census data and used tree-based, gradient-boosted regression models to identify clinical and socioeconomic predictors of resource use. Separate analyses of cumulative numbers of hospital days and ED visits modeled the probability of any resource use and, when present, the amount of resource use. We used relative importance in each analysis to identify important predictors.

**Results:**

The analytic sample included 11 105 children with medical complexity. The best short- and long-term predictors of having a hospital stay and number of hospital days were initial length of stay and clinical classification. Initial length of stay, residence rurality, and other socioeconomic factors were top predictors of short-term ED use. The top predictors of ED use in the long term were almost exclusively socioeconomic, with rurality a top predictor of number of ED visits. Estimates of marginal associations between initial length of stay and resource use showed that average number of hospital days increases as initial length of stay increases up to approximately 90 days. Children with medical complexity living in rural areas had more ED visits on average than those living in urban or metropolitan areas.

**Conclusions:**

Clinical factors are generally better predictors of hospital use whereas socioeconomic factors are more predictive of ED use among children with medical complexity in Alberta. The results confirm existing literature on the importance of socioeconomic factors with respect to health care use by children with medical complexity.

## Introduction

Children with medical complexity (CMCs) are characterized as having at least 1 complex chronic condition (CCC) that requires significant health care support or medical technology for daily living [1]. Although relatively few in number (about 1% of children), studies from the United States, Australia, and Canada have reported that CMCs account for a substantial amount of pediatric hospitalizations [2–4]. Specifically in Canada, the Canadian Institute for Health Information (CIHI) reported that CMCs accounted for 57% of hospital care costs in 2015–2016 among children and youth [4]. CMCs are also twice as likely to access the emergency department (ED) than non-CMC children, more likely to return to the ED after being discharged, and more likely to be admitted [4].

A robust understanding of health care utilization trends, notably of hospital and ED use [5], among CMCs over time supports the development of more-effective policies, the optimization of care delivery, and the enhancement of caregiver support through the development of models of care delivery [6]. In a study to establish research priorities for children and youth with special health care needs, Coller et al. [7] identified several important factors, including social determinants of health, family support programs, and clinical care delivery. All these factors interact in complex ways over time, which can be challenging to model. Longitudinal studies are particularly important for the CMC population as health care needs and access to health care services change over time [8].

Much of the research on CMCs to date has focused on understanding associations between clinical factors and health care resources and consider only a limited number of socioeconomic factors. Socioeconomic factors play an important role in the health of children and are even more important for CMCs [9]. In general, the use of health care services such as hospitals and EDs depends on many factors that include the socioeconomic status of families such as location of residence and the availability of financial and community support [10]. However, there is a lack of administrative health data linked with socioeconomic data within Electronic Health Records (EHRs) to encourage and facilitate research in this area [11].

Both inferential and predictive models can be used to describe associations between clinical and socioeconomic factors and health care use over time. However, health care resource use for CMCs has historically only been described in terms of population-level trends, typically under traditional inferential regression frameworks [12] that are limited in terms of their complexity and predictive capacity. Predictive models, such as those common in machine learning, are better able to handle complex interaction structures and report on the predictive importance of large numbers of variables [12]. The lack of predictive modeling in CMC research represents a significant gap in the literature.

Despite its increasing accessibility, modern machine learning methods have seen slow adoption in CMC-related research. Applications of machine learning in the health care literature more generally have typically been limited to the diagnosis, screening, and prediction of outcomes for a small number of specific diseases such as cancers, nervous system diseases, or cardiovascular diseases [13]. As CMCs are a highly heterogeneous population with diverse clinical and socioeconomic characteristics, it is critical to accommodate the many interaction effects relevant to this group [14]. Machine learning models in the study of CMCs thus have the potential to yield valuable insights and drive further research.

However, predictive modeling for CMCs has arguably been slowed by the availability and composition of administrative health care data. In this sense, the design of EHRs has the potential to accelerate or bottleneck research in this field [15]. The marriage of EHRs and predictive modeling is an emerging area of innovation in health care that can improve patient outcomes and decision-making processes [16,17]. Developments in this area are thus also broadly relevant to jurisdictions developing EHRs (eg, Connect Care in Alberta) in addition to CMC-related research specifically.

The purpose of this study was to identify and compare important predictors of short- and long-term health care resource use (ie, hospital days and ED visits) at the individual level among CMCs in Alberta, Canada. A secondary objective was to estimate marginal associations for the most important predictors for further insight. Our results motivate, as another secondary objective, further discussion of the utility of administrative data in CMC-related research and how EHRs can be designed to facilitate predictive analyses in the future for CMC populations.

## Materials and methods

This retrospective, population-cohort study links administrative health data to socioeconomic data from the Canadian census to identify clinical and socioeconomic predictors of hospital and ED visits for CMCs in Alberta. The University of Alberta Research Ethics Board (Pro00103550_REN2) granted research ethics approval for this study on December 7^th^, 2020. Data was de-identified before authors obtained access on April 26^th^, 2021.

### Study design and data

The Maternal Newborn Child Youth Strategic Clinical Network under Alberta Health Services (AHS) extracted data for this study from health administrative datasets housed in the AHS data repository (AHSDR). The AHSDR houses the Discharge Abstract Database (DAD), which contains hospital discharge data [18]; the National Ambulatory Care Reporting System (NACRS) [19], which contains data on hospital ED visits; Alberta Vital Statistics [20], which lists all births and deaths in Alberta; and the AHS Pharmaceutical Information Network (PIN) [21], which contains data on pharmaceuticals dispensed in a community setting.

### Study population and primary outcome variables

CMCs were identified using DAD via an ICD-10-based definition commonly used in the literature [22]. CMCs were included if they were Alberta residents, were at most 18 years old at admission, and had an initial discharge and diagnosis between 2010 and 2013. The primary outcome variables were annual cumulative number of hospital days and ED visits (at the patient level) for the first and fifth years immediately following an initial hospital discharge (first admission and discharge with a CCC diagnosis). These outcomes were obtained from DAD and NACRS.

### Study variables

Demographic data included patients’ age and sex. Clinical information included initial hospitalization length of stay (LOS) in days, number of chronic medications 1 year prior to initial admission (from PIN data), clinical classification (ie, single vs multiple complex chronic conditions), 9 indicators for chronic disease involving different body systems (from DAD and NACRS data), the presence of technology assistance (eg, home oxygen), and 7-day and 30-day readmission following initial discharge. The presence of technology assistance utilized procedure codes established by Cohen et al. [22]. All variables were measured at initial admission unless otherwise specified.

Socioeconomic variables were obtained through residential postal codes collected at initial discharge and included AHS zone, residence rurality, material and social deprivation scores, Canadian census data, and (from DAD and NACRS) mother use of mental health services in the 12 months prior to their child’s initial admission. AHS has 5 zones (North, Edmonton, Central, Calgary, and South) and classifies regions of Alberta into 6 rurality categories ranging from metropolitan to remote rural. AHS zones are geographic areas with operational accountability to deliver health care services within an integrated provincial health system. Material and social deprivation were measured through the Pampalon index based on 6 variables from Canadian census data measured at the dissemination area (DA) level [23]. These 6 component variables, which include average household income as well as proportions of single-parent families; individuals who are divorced, widowed, or separated; individuals living alone; unemployed individuals; and individuals without a high school diploma were also included.

### Analysis

We used tree-based, gradient-boosted regression models to predict each of the 2 primary outcomes (cumulative hospital days and cumulative ED visits) after 1 and 5 years following initial discharge using clinical and socioeconomic factors. Gradient boosting with regression trees is well established in the statistical literature [24] and is conceptually similar to random forests: both frameworks obtain a sequence of decision trees that are used in tandem to predict a response. Unlike random forests, which grow trees independently, gradient boosting models grow trees that successively improve upon the previous trees. For a gentle introduction to tree-based methods, see King & Strumpf [12].

Each of the 4 models contained 2 submodels: the first predicted the presence of hospital days or ED visits (in a binary model), while the second predicted number of hospital days among CMCs with nonzero resource use (in a conditional model). Together, these 2 submodels fit into a more-general hurdle model framework. Linear hurdle regression models have previously been used in studies of health care resource use and expenditure [25], also specifically for CMCs [26]. We identified important predictors of hospital days and ED visits in the first 1 and 5 years following an initial admission through the relative importance of each variable. This summary measure describes the proportion of variance explained by each predictor included in the model (out the total amount of variance explained by the model) [24].

Relative importance measures the contribution of each predictor variable to overall model performance (measured as a percentage between 0% and 100%). Predictors that are very useful in predicting the response have a high importance, while predictors that only marginally improve prediction accuracy have a low importance. It is calculated for each predictor in a model by measuring the degradation in predictive performance when the observed values of that variable are randomly permuted. Relative importance is scaled so that the sum of the relative importance of all variables in the model is always equal to 100%.

We included as predictors sex, age group, clinical conditions (using age category and ICD-codes based on Cohen et al. [22] and Feudtner et al. [27]), technology assistance, number of chronic medications, readmission indicators, residence rurality, mother mental health service use (with missing data treated as a separate categorical level, “no data available”), initial LOS, material and social deprivation scores derived from the Pampalon index, and the 6 DA-level measures described in the previous section. The clinical factors included are proxies for clinical complexity, while socioeconomic factors are generally known to be associated with health resource use and are important to consider together with clinical factors [27].

All analyses were performed with R (version 3.6.3) [28] using gbm (package version 2.1.8.1) [29]. We separately tuned the hyperparameters (namely, number of trees, tree height, shrinkage, and terminal node size) of each model via caret (version 6.0–93) [30] by iteratively refining a coarse hyperparameter grid to maximize prediction accuracy (of the binary submodels) or to minimize root mean-squared error (of the conditional submodels) via 5-fold cross-validation. We applied a log transformation to both responses in the conditional submodels to reduce the effect of outliers.

We used a training set of 90% of the cohort’s CMCs to train the models and estimated generalization error using a hold-out testing set of the remaining 10%. We summarized binary and conditional model performance on both sets with AUC (ie, area under the ROC curve) and R^2^ (ie, proportion of response variability explained), respectively. AUC is interpretable as the probability that a model will be able to correctly differentiate between a randomly selected (case/non-case) pair of subjects. In a secondary analysis, we presented estimates of marginal associations for select important variables. These estimates were calculated by integrating out all other variables, as described in [24]. All confidence intervals were obtained using a simple nonparametric bootstrap (with 100 bootstrap samples). To assess model fit, we used calibration curves for the binary model (to assess predicted probabilities of a non-zero response) and for the conditional model we examined plots of true vs. predicted values.

## Results

### Cohort characteristics

In total, 12 621 CMCs were included in the sample. Over half of the sample were newborns (<1 year old) at initial admission. Of the cohort, 82.3% had 1 CCC, 13.8% had 2, and 3.9% had 3 or more. Number of chronic medications was most commonly 0 (79.5%), 1 (9.4%), or 2–4 (8.8%). Most CMCs lived in metropolitan areas (64.6%) and a minority lived in rural (19.5%) or remote rural (4.3%) areas. See Table 1 for detailed cohort characteristics. In the first year following initial discharge, 4611 (41.5%) and 6385 (57.5%) CMCs had at least one hospital day and ED visit, respectively; among these CMCs, the median (Q_1_, Q_3_) number of hospital days and ED visits was 7.0 (2.0, 23.0) and 2.0 (1.0, 4.0), respectively. In the first five years following initial discharge, 5943 (53.5%) and 9262 (83.4%) CMCs had at least one hospital day and ED visit, respectively; among these CMCs, the median (Q1, Q3) number of hospital days and ED visits was 9.0 (3.0, 29.0) and 4.0 (2.0, 9.0), respectively.

**Table 1:** CMC cohort characteristics (n = 12 621).

Characteristic	Analytic sample, no. (%) (n = 11 105)	Excluded patients, no. (%) (n = 1516)	*P* value^a^
Female^b^	5105 (48.6)	716 (47.2)	.36
Age^c^, years			
Median (Q_1_, Q_3_)	0.0 (0.0, 9.0)	0.0 (0.0, 8.0)	.71
Newborns (<1)	5699 (51.3)	766 (50.5)	.58
Clinical group^c^^,^^d^			
Cardiology	3438 (31.0)	488 (32.2)	.35
Neurology	3165 (28.5)	441 (29.1)	.66
Malignancy	1531 (13.8)	184 (12.1)	.09
Congenital/genetic	1347 (12.1)	177 (11.7)	.64
Respiratory	1125 (10.1)	152 (10.0)	.94
Renal	1016 (9.1)	126 (8.3)	.31
Gastrointestinal	630 (5.7)	93 (8.3)	.51
Metabolic	589 (5.3)	92 (6.1)	.24
Hematology/immunodeficiency	291 (2.6)	44 (2.9)	.58
Technology assistance	461 (4.2)	69 (4.6)	.51
Number of chronic medications^e^			.14
0	8844 (79.6)	1187 (78.3)	
1	1039 (9.4)	143 (9.4)	
2–4	980 (8.8)	139 (9.2)	
≥5	242 (2.2)	47 (3.1)	
Initial LOS, days, Median (Q_1_, Q_3_)	3.0 (1.0, 8.0)	3.0 (1.0, 8.0)	.42
Residence rurality^c^^,^^f^			< .001
Metropolitan	7172 (64.6)	237 (15.6)	
Urban	1292 (11.6)	66 (4.4)	
Rural	2164 (19.5)	196 (12.9)	
Remote rural	477 (4.3)	2 (0.1)	
Missing^g^	0 (0.0)	1015 (67.0)	
Mother use of MHS^e^			.82
Accessed MHS	1904 (17.1)	253 (16.7)	
Did not access MHS	5482 (49.4)	761 (50.2)	
Data unavailable	3719 (33.5)	502 (33.1)	
DA-level summaries,^h^^,^^i^ median (Q_1_, Q_3_)			
Material deprivation	0.01 (-0.02, 0.04)	-	-
Social deprivation	0.00 (-0.02, 0.03)	-	-
Single-parent families	0.14 (0.10, 0.21)	0.15 (0.09, 0.21)	.52
Separated, divorced or widowed^j^	0.17 (0.13, 0.22)	0.21 (0.15, 0.27)	< .001
Living alone^j^	0.07 (0.04, 0.12)	0.11 (0.07, 0.19)	< .001
Income,^i^ CAD 1000	44.6 (36.0, 48.2)	40.8 (34.7, 53.6)	< .001
Employed^j^	0.70 (0.62, 0.76)	0.67 (0.55, 0.74)	< .001
No high-school diploma^j^	0.18 (0.12, 0.26)	0.22 (0.13, 0.33)	< .001

Abbreviations: CAD, Canadian dollars; CMC, child with medical complexity; DA, dissemination area; LOS, length of stay; MHS, mental health services.

^a^ Comparisons were conducted via Wilcoxon signed-rank tests (for age, initial LOS, and DA-level summaries), 2-sample tests for proportions (for sex, proportion of newborns, and clinical groups), Pearson’s chi-squared tests (for number of chronic medications, rurality, and MHS use). P values are reported without correction.

^b^ All other CMCs were male, with the exception of 1 with a noted sex of “Other”.

^c^ At initial admission.

^d^ A CMC may fall into more than 1 clinical group, so percentages need not add to 100%.

^e^ In the 12 months prior to a CMC’s initial admission.

^f^ From the original dataset, “moderate metropolitan influence”, “moderate urban influence”, and “rural center area” were merged into “metropolitan”, “urban”, and “rural”, respectively.

^g^ Excluded from the comparison

^h^ Summary of DA-level proportions (or averages, for income) across the sample.

^i^ Calculated for the excluded sample where data was available. Deprivation scores were available for only 1 CMC in the excluded sample, so summaries and comparisons are omitted here.

^j^ Among individuals in a DA at least 15 years of age.

We excluded 1516 CMCs (12.0%) who were missing socioeconomic data (1515) or had a sex of “other” (1). The excluded CMCs were comparable to the remaining 11 105 CMCs in the analytic cohort except on the basis of 5 of the DA-level measures (Table 1).

## Performance

On the training set and across both periods and primary outcomes, AUC for the binary models was moderate–high between 0.70 and 0.78. On the testing set, AUC was moderate [31] at about 0.61 for the ED visit models and 0.71 for the hospital day models. Performance was poorer for the conditional submodels, where R^2^ was low, falling between 0.10 and 0.29, and was generally lower in year 5. See S1 Table for exact performance measures.

## Predictor importance

**Fig 1:**
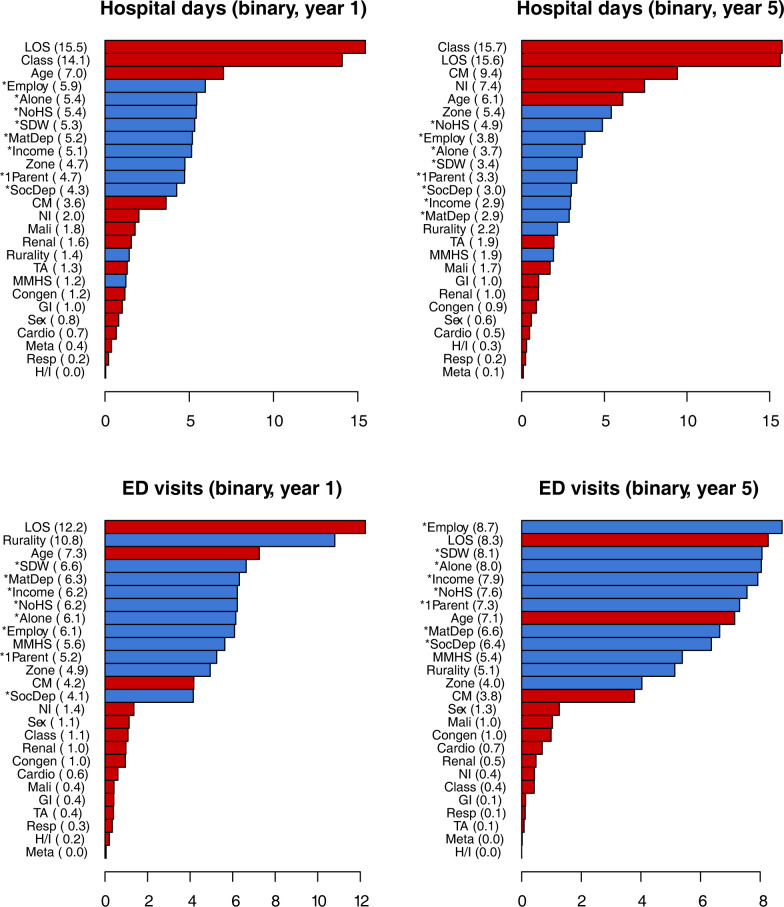
Relative variable importances for the binary submodels. Relative importance is given in parentheses. Clinical and socioeconomic variables are indicated with red and blue, respectively. An asterisk (*) denotes a DA-level measure. Abbreviations: 1Parent, proportion of single-parent families; Alone, proportion of individuals living alone; Cardio, cardiology condition indicator; Class, single or multiple complex chronic conditions; CM, number of chronic medications; Congen, congenital/genetic condition indicator; Employ, employment rate; GI, gastrointestinal condition indicator; H/I, hematology/immunodeficiency condition indicator; MatDep, material deprivation score; Mali, malignancy condition indicator; Meta, metabolic condition indicator; MMHS, mother mental health service use; NI, neurological impairment indicator; NoHS, proportion of individuals without a high school diploma; Renal, renal condition indicator; Resp, respiratory condition indicator; SDW, proportion of individuals who are separated, divorced, or widowed; SocDep, social deprivation score; TA, technology assistance indicator.

**Fig 2:**
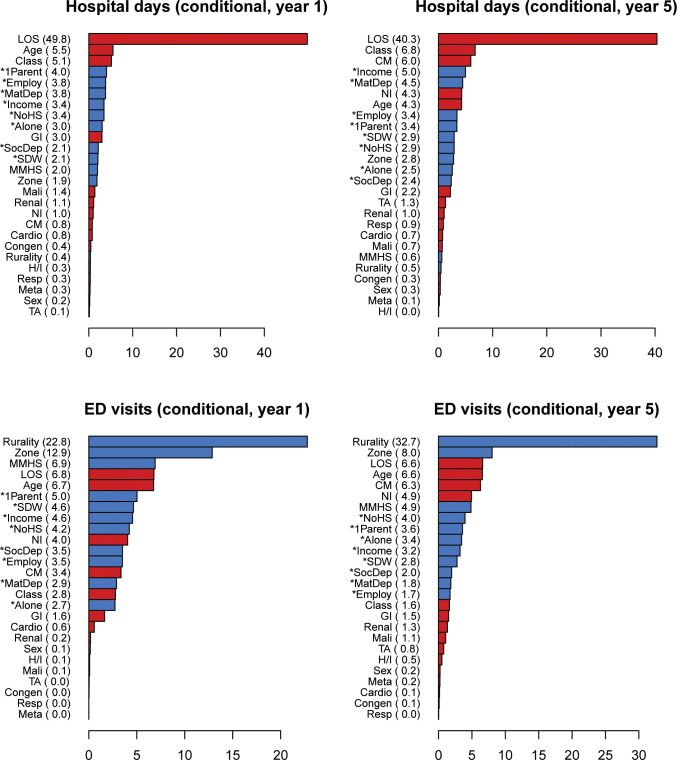
Relative variable importances for the conditional submodels. Relative importance is given in parentheses. Clinical and socioeconomic variables are indicated with red and blue, respectively. An asterisk (*) denotes a DA-level measure. Abbreviations: 1Parent, proportion of single-parent families; Alone, proportion of individuals living alone; Cardio, cardiology condition indicator; Class, single or multiple complex chronic conditions; CM, number of chronic medications; Congen, congenital/genetic condition indicator; Employ, employment rate; GI, gastrointestinal condition indicator; H/I, hematology/immunodeficiency condition indicator; MatDep, material deprivation score; Mali, malignancy condition indicator; Meta, metabolic condition indicator; MMHS, mother mental health service use; NI, neurological impairment indicator; NoHS, proportion of individuals without a high school diploma; Renal, renal condition indicator; Resp, respiratory condition indicator; SDW, proportion of individuals who are separated, divorced, or widowed; SocDep, social deprivation score; TA, technology assistance indicator.

Refer to Figs 1 and 2 for individual variable importances in the binary and conditional submodels, respectively.

### Short-term predictors

The best predictors of having a hospital stay within the first year were initial LOS and clinical classification, which were at least 2–3 times as important as age (the third most important predictor) and the other socioeconomic variables. Among CMCs with hospital days in the first year, initial LOS was by far the most important predictor of number of hospital days, with an importance at least 9 times higher than age and clinical classification (the second and third most important predictors) and the socioeconomic variables.

Initial LOS was also the most important predictor of ED visits within the first year, but was close in importance to residence rurality and about 1.5–2.5 times as important as age (the third most important predictor) and the other socioeconomic factors. Among CMCs with ED visits in the first year, however, socioeconomic variables (namely rurality, AHS zone, and mental health service use) were the most important predictors of number of ED visits. In particular, rurality was at least 3 times as important as initial LOS and age, the most important clinical/demographic variables.

### Long-term predictors

Clinical classification and initial LOS had similar importances and were top predictors of the presence of a hospital stay within 5 years. Both were at least 2 times as important as any socioeconomic factor. Number of chronic medications, the presence of neurological impairment, and age were the third through fifth most important predictors. Similar to the short-term results, initial LOS was by far the most important predictor of number of hospital days (among CMCs with a hospital stay within the 5 years), and was at least 5.5 times as important as clinical classification and number of chronic medications (the next most important predictors) and any other socioeconomic factor.

The top 10 predictors of the presence of ED visits were comparable in terms of relative importance. All but 2 (initial LOS and age) of these predictors were socioeconomic factors. Among CMCs with ED visits within 5 years, residence rurality was at least 4 times as important as every other predictor.

## Marginal associations

### Short-term predictors

**Fig 3:**
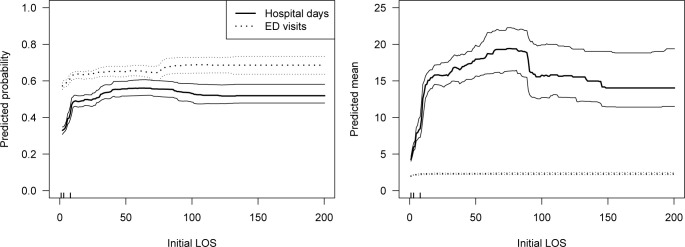
Marginal associations (and 95% confidence intervals) for initial LOS in the binary (left) and conditional (right) submodels in the year following initial discharge. Estimates for hospital days are indicated with solid lines and those for ED visits with dotted lines. The rugs on the horizontal axes indicate the first through third quartiles of initial LOS in the analytic sample (given numerically in Table 1). Abbreviations: ED, emergency department; LOS, length of stay.

Fig 3 shows marginal association estimates for initial LOS, a consistently important predictor of hospital days. Among CMCs with a hospital stay, the mean number of hospital days in year 1 increased from about 5 to 20 days as initial LOS increased to 90 days, and afterwards decreased and plateaus (although the wide confidence intervals suggests that a plateau at around 45 days is also plausible). The marginal association between initial LOS and the probability of a hospital stay was similar and increased (from about 0.30 to 0.55) with initial LOS up to 50 days.

**Fig 4:**
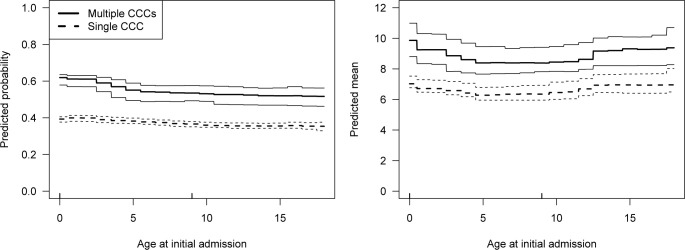
Marginal associations (and 95% confidence intervals) for age at initial admission in the binary (left) and conditional (right) submodels for hospital days in the year following initial discharge. Estimates for CMCs with multiple CCCs are indicated with solid lines and those for a single CCC with dotted lines. The rugs on the horizontal axes indicate the first through third quartiles of age at initial admission in the analytic sample (given numerically in Table 1). Abbreviations: CCC, complex chronic condition; CMC, child with medical complexity.

As shown in Fig 4, younger patients were slightly more likely to be hospitalized than older patients in the first year. This was particularly true for CMCs with multiple CCCs. The marginal association between age and the probability of having an ED visit was similar but was nearly zero in the conditional submodel for number of ED visits (S1 Fig).

Relative to CMCs with a single CCC, those with multiple CCCs were more likely to have hospital days (0.59, 95% CI 0.55–0.60 vs 0.38, 95% CI 0.36–0.39) in the first year. Similarly, among CMCs with hospital days in the first year, those with multiple CCCs had more hospital days on average (9.37, 95% CI 8.49–6.61 vs 6.85, 95% CI 6.61–7.28) than those with a single CCC.

CMCs in remote rural (0.67, 95% CI 0.64–0.70) areas were the most likely to have an ED visit in the first year, followed by those in rural (0.67, 95% CI 0.64–0.68), urban (0.55, 95% CI 0.54–0.57), and metropolitan (0.55, 95% CI 0.54–0.56) areas. Similarly, among CMCs who had ED visits, those in remote rural (2.62, 95% CI 2.45–2.80) areas had the most visits on average, followed by those in rural (2.56, 95% CI 2.45–2.80), metropolitan (2.00, 95% CI 1.94–2.04), and urban (1.99, 95% CI 1.93–2.05) areas. See S2 Table for estimates by AHS zone and rurality, which are consistent with the above marginal estimates.

Marginally, among CMCs with ED visits in the first year, those whose mothers had used mental health services had a higher number of ED visits relative to those whose mothers did not use these services (2.42, 95% CI 2.31–2.52 vs 2.09, 95% CI 2.04–2.13). Estimates where data on service use was unavailable were comparable to those for the latter group (2.09, 95% CI 2.04–2.14).

### Long-term predictors

The marginal effects of initial LOS on 5-year outcomes were similar to those for the first year (S2 Fig).

As in the first year, CMCs with multiple CCCs were more likely to have hospital days than CMCs with a single CCC (0.68, 95% CI 0.65–0.70 vs 0.51, 95% CI 0.50–0.52). Among CMCs with hospital days, those with multiple CCCs also had more hospital days on average than those with a single CCC (12.7, 95% CI 11.38–13.83 vs 8.54, 95% CI 8.15–8.89).

Marginally, CMCs with more chronic medications were more likely to have a hospital stay within the 5 years (0.70, 95% CI 0.63–0.73 for ≥5 medications; 0.62, 95% CI 0.58–0.65 for 2–4 medications; 0.58, 95% CI 0.55–0.61 for 1 medication; 0.52, 95% CI 0.51–0.53 for 0 medications). Similarly, among CMCs with hospital days, those with more chronic medications had more on average (15.15, 95% CI 12.37–17.71 for ≥5 medications; 10.93, 95% CI 9.70–11.93 for 2–4 medications; 9.92, 95% CI 9.14–10.77 for 1 medication; 8.76, 95% CI 8.44–9.20 for 0 medications).

While employment rate (at the DA level) was the most important predictor of the presence of ED visits by the fifth year, its marginal effect was close to zero (S3 Fig). Similarly, while marital status (namely, the proportion of individuals in a DA who are separated, divorced, or widowed) was the third most-important predictor of the presence of hospital days, its marginal effect was also plausibly zero (S4 Fig).

Similar to short-term outcomes, CMCs in remote rural (0.88, 95% CI 0.86–0.88) and rural (0.88, 95% CI 0.86–0.89) areas were the most likely to have an ED visit within the 5 years, followed by those in urban (0.83, 95% CI 0.82–0.85) and metropolitan (0.83, 95% CI 0.82–0.84) areas. Similarly, among CMCs who had ED visits, those in remote rural (6.16, 95% CI 5.70–6.61) areas had the most on average, followed by those in rural (6.04, 95% CI 5.74–6.28), metropolitan (3.96, 95% CI 3.86–4.07), and urban (3.96, 95% CI 3.84–4.12) areas.

## Discussion

To our knowledge, this is the first study to develop a machine learning model to identify predictors of hospital days and ED visits among CMCs. We used a typical curated administrative dataset containing proxies for both clinical complexity and socioeconomic status. Generally, and for both short- and long-term outcomes, clinical variables were the most important predictors of (the presence and number of) hospital days while socioeconomic variables (despite some being DA-level proxies for household-level measures) were the most important predictors of ED visits. Our results are consistent with the Andersen behavioral model [10] of access to health care resources, specifically regarding the importance of socioeconomic and demographic characteristics beyond health care availability.

### Clinical variables

As might be expected from proxies for clinical complexity, initial LOS and clinical classification (ie, single vs multiple CCCs) were top predictors of hospital use. The generally consistent importance of initial LOS as a predictor of future resource use should prompt further investigation of factors related to initial LOS, including the collection of more-nuanced data from the initial hospitalization event (eg, procedures performed, medications administered, physician assessment notes, etc). Understanding specific risk factors that are related to initial LOS and are predictive of subsequent hospital and ED utilization would support service planning and the development of care pathways.

Similarly, the importance of clinical classification and number of chronic medications as predictors of hospital use could suggest that CMCs with multiple CCCs or a large number of chronic medications (at initial admission) be provided additional supports beyond the first year in response to elevated projections for long-term resource use.

Differences in the patterns of importance between the short- and long-term models for hospital stays and ED visits suggested that individual clinical indicators were typically less useful as predictors than the other variables considered. However, the neurology clinical indicator was at least 4–5 times as important as the other clinical indicators in predicting the number of hospital days and ED visits over the 5 years among CMCs who used these resources. This result suggests that the subpopulation of CMCs with neurological conditions are more vulnerable and may require increased health care resources relative to other CMCs which is consistent with other studies in the literature [32–34].

### Socioeconomic variables

CMCs in rural and remote rural locations as well as those in the “mainly rural” AHS zones had the highest probability of an ED visit. This could be due to several reasons, including unmet care or the availability of after-hours care or a medical home. This finding is consistent with a U.S. study by Barnert et al. [35], who reported that socioeconomic factors such as geographic location and economic position are associated with access to care and parental stress for CMCs and their families.

The finding that CMCs whose mothers accessed mental health services (prior to their child’s initial admission) were more likely to have ED visits in the first year prompts further investigation. It is possible that mothers with a history of accessing mental health services are less able to manage their child’s illness and thus require more support [36]. Conversely, it is also possible that mothers who access the health system for themselves are more likely to be able to navigate and use health care resources for their child. Since we do not have the data to understand what services these mothers are utilizing, we must leave delineating the exact nature of this relationship to future work.

Speaking to ED visits specifically, the most important predictors of long-term resource use (except initial LOS) were socioeconomic in nature. From the relatively high importance of employment status and marital status as predictors of the presence of ED visits, we hypothesize that family structure, residence rurality, and financial resources are important to CMC outcomes. The near-zero marginal associations of these and other socioeconomic variables (not shown in this work) such as living alone, income, education, single parenting, material and social deprivation, and maternal mental health access, however, suggests that their importance is “hidden” in higher-order interactions with other variables. The complexity of these interactions makes it difficult to directly identify policies improving CMC and family outcomes without further specific research.

These results confirm the importance of population characteristics as described in the Anderson model [10]: socioeconomic factors such as financial resources and residence location together with health care policies influence how CMCs and families use health care resources. For example, Alberta has 2 large pediatric hospitals located in large metropolitan cities. It is possible that CMCs who live in (remote) rural locations have higher ED use due to unequal access to health care resources and supports such as medical homes or care-coordination services, and that this inequity makes EDs the most feasible option for CMC families. It is possible that different policies and processes in rural care delivery contribute to this inequity. For example, in rural Alberta, primary care physicians provide service in the local emergency department, which potentially reduces available clinic hours. According to Hudson [33], there are few studies examining hospital readmission and ED use for children with special health care needs in rural and community settings relative to regional centers.

Lastly, the marginal effect of age showed that younger CMCs with multiple CCCs were more likely to be hospitalized than patients diagnosed at an older age. This finding is consistent with Agrawal et al. [34], who showed that the top 5% of child health care users on Medicaid did not remain in the top 5% in subsequent years, indicating reduced health care resource needs over time. This is an important consideration since about half of our cohort was less than 1 year old at initial admission. Anticipating changes in health care resource needs for younger CMCs over time can aid in effective policy and care delivery programs.

### Machine learning and health administrative data

Beyond existing applications in health care to improve diagnostics and identify risk factors for various diseases [35], there is substantial potential for machine learning in health care. This includes supporting clinical decision-making [37], identifying risk factors within patient populations [16,38], supporting research in epidemiology [15], and identifying factors affecting health care resource use [39]. Machine learning models, relative to traditional statistical methods such as regression, may be superior for predictive modeling in heterogeneous, clinically complex populations such as CMCs because they are better equipped to handle high-dimensional data and complex interactions [12]. However, the content of administrative health data, including those collected in existing EHRs, may be limiting this potential. EHRs should be designed to facilitate and encourage predictive modeling by collecting diverse, detailed, patient-level characteristics. Patient-level measures such as income level, family structure or parental access to respite care are all examples of socioeconomic data that can be included in EHRs. Clinical notes such as home-care nursing visits, structured assessment notes (beyond ICD-10 codes) can also be included in support of this goal but are substantially more complicated to analyze. Early evidence suggests that the use of predictive modeling with well-designed EHR systems can improve patient outcomes and clinical decision making [17].

This study examined both clinical and socioeconomic variables and showed that the latter were particularly important predictors of (binary) ED utilization in the short and long terms. The performance of our conditional models suggested a high amount of unexplained variability in both the number of hospital days and ED visits. We originally observed this unexplained variability in a set of random forest models (not presented here) trained on the same data. Despite the relatively greater capacity and expressiveness of the boosted models presented in this work, we saw little improvement in the performance of the conditional models. While this reflects the fact that forecasting resource use for CMCs is an inherently difficult task [40], it may also suggest that there is insufficient signal in the administrative data used in this analysis. These findings motivate our previous calls for more-robust clinical and socioeconomic data in EHRs in order to better support predictive modeling for CMC populations in the future. Socioeconomic factors are particularly important to consider for the CMC population because the families of CMCs struggle with economic and psychosocial effects that impact the child, family health, and subsequent use of health care resources [41,42].

## Limitations

This study has limitations common to retrospective research with curated administrative data. First, the socioeconomic data linked to our clinical data represent DA-level characteristics, which do not accurately reflect individual- or household-specific variability. Second, the independent variables in this study were only available around initial admission or discharge, while time-varying predictors (ie, varying across the 5 years) may yield more-interpretable and more-practical models. Third, while ICD codes are well defined and readily used in administrative health data to identify CMCs, they lack detail regarding the medical complexity of the patient, which can further contribute to a reduced ability to represent individual CMCs with available data. Another consideration for interpreting socioeconomic predictors such as rurality and AHS Zone is that the availability of primary care services was not available in the dataset and uneven distribution of pediatric specialty services (eg, large pediatric centers are available in Edmonton and Calgary Zones but not the rest of the province) may be confounding factors.

As discussed above, finer patient-level data such as details of the initial LOS encounter, patient-level socioeconomic factors, and names of chronic medications prescribed may be necessary to explain the large amount of variability in resource use observed in this study. This is in line with our call for EHRs to collect more-detailed patient-level data, particularly that related to socioeconomic conditions. However, future work is necessary to examine practical aspects of EHR design and burdens associated with data collection.

## Conclusions

This study used a machine learning model and readily available administrative health care data to identify important predictors of hospital days and ED visits, in both the short and long terms, among CMCs in Alberta, Canada. Initial LOS and clinical classification, factors indicative of medical complexity, were strong predictors of hospital days. Residence rurality, socioeconomic metrics, and initial LOS were predictive of ED use. Our results are in line with existing literature and suggest the necessity of further examinations of the impact of residence rurality and how an initial hospitalization event influences outcomes for CMCs and their families. We encourage health care systems to actively design data collection systems and EHR processes that support robust analyses and the development of high-accuracy predictive models for the highly heterogeneous CMC population.

## Supporting information

S1 Table Model performance on the training and testing sets: AUC and R^2^ for the binary and conditional submodels. (PDF)

S2 Table Estimated mean conditional number of ED visits (with 95% confidence intervals) by AHS zone and residence rurality. (PDF)

S1 Fig Marginal associations (and 95% confidence intervals) for age at initial admission in the binary (left) and conditional (right) submodels for ED visits in the year following initial discharge.
(TIF)

S2 Fig Marginal associations (and 95% confidence intervals) for initial LOS in the binary (left) and conditional (right) submodels in the fifth year following initial discharge.
(TIF)

S3 Fig Marginal associations (and 95% confidence intervals) for employment rate (measured at the DA level) in the binary (left) and conditional (right) submodels in the fifth year following initial discharge.
(TIF)

S4 Fig Marginal associations (and 95% confidence intervals) for the proportion of single, divorced, or widowed individuals (measured at the DA level) in the binary (left) and conditional (right) submodels in the fifth year following initial discharge.
(TIF)
