# Supplementary material for: Clinical and socioeconomic predictors of hospital use and emergency department visits among children with medical complexity: A machine learning approach using administrative data
Source: PLoS One. 2024 Oct 29;19(10):e0312195. doi: 10.1371/journal.pone.0312195 (PMC11521260; doi:10.1371/journal.pone.0312195)
Supplement: S1 Table — (PDF) [file pone.0312195.s001.pdf]

S1 Table. Model performance on the training and testing sets: AUC and R^2 for the binary and conditional submodels.

Year	Outcome	Binary model performance, AUC		Conditional model performance, R^2	
		Training	Testing	Training	Testing
Year 1	Hospital days	0.75	0.71	0.29	0.18
	ED visits	0.70	0.61	0.24	0.19
Year 5	Hospital days	0.73	0.72	0.13	0.10
	ED visits	0.78	0.62	0.14	0.10

Abbreviations: AUC, area under the ROC curve; ED, emergency department; ROC, receiver–operator characteristic.
