# Supplementary material for: Clinical and socioeconomic predictors of hospital use and emergency department visits among children with medical complexity: A machine learning approach using administrative data
Source: PLoS One. 2024 Oct 29;19(10):e0312195. doi: 10.1371/journal.pone.0312195 (PMC11521260; doi:10.1371/journal.pone.0312195)
Supplement: S2 Table — (PDF) [file pone.0312195.s002.pdf]

S2 Table. Estimated mean conditional number of ED visits (with 95% confidence intervals) by AHS zone and residence rurality.

AHS zone	Year	Residence rurality			
		Metropolitan	Urban	Rural	Rural remote
Calgary	1	1.87 (1.83, 1.93)	-	2.37 (2.22, 2.52)	-
	5	3.80 (3.71, 3.91)	-	5.74 (5.42, 5.99)	-
Central	1	2.03 (1.90, 2.13)	-	2.57 (2.40, 2.75)	-
	5	3.85 (3.74, 4.08)	-	5.83 (5.50, 6.17)	-
Edmonton	1	1.96 (1.88, 2.03)	-	-	-
	5	3.86 (3.75, 4.01)	-	-	-
North	1	-	2.33 (2.20, 2.49)	3.05 (2.84, 3.20)	3.11 (2.85, 3.31)
	5	-	4.70 (4.40, 5.01)	7.27 (6.77, 7.73)	7.40 (6.77, 8.00)
South	1	-	1.96 (1.87, 2.09)	2.53 (2.34, 2.67)	-
	5	-	3.95 (3.77, 4.25)	6.00 (5.63, 6.53)	-

Abbreviation: AHS, Alberta Health Services; ED, emergency department.

Note: A - indicates estimates omitted due to the composition of a zone (eg, no remote rural areas exist in the Calgary Zone) or the small number of CMCs in the cohort (i.e., <50 in urban Central, rural Edmonton, and metropolitan
