# Supplementary figures and images for: Clinical and socioeconomic predictors of hospital use and emergency department visits among children with medical complexity: A machine learning approach using administrative data
Source: PLoS One. 2024 Oct 29;19(10):e0312195. doi: 10.1371/journal.pone.0312195 (PMC11521260; doi:10.1371/journal.pone.0312195)

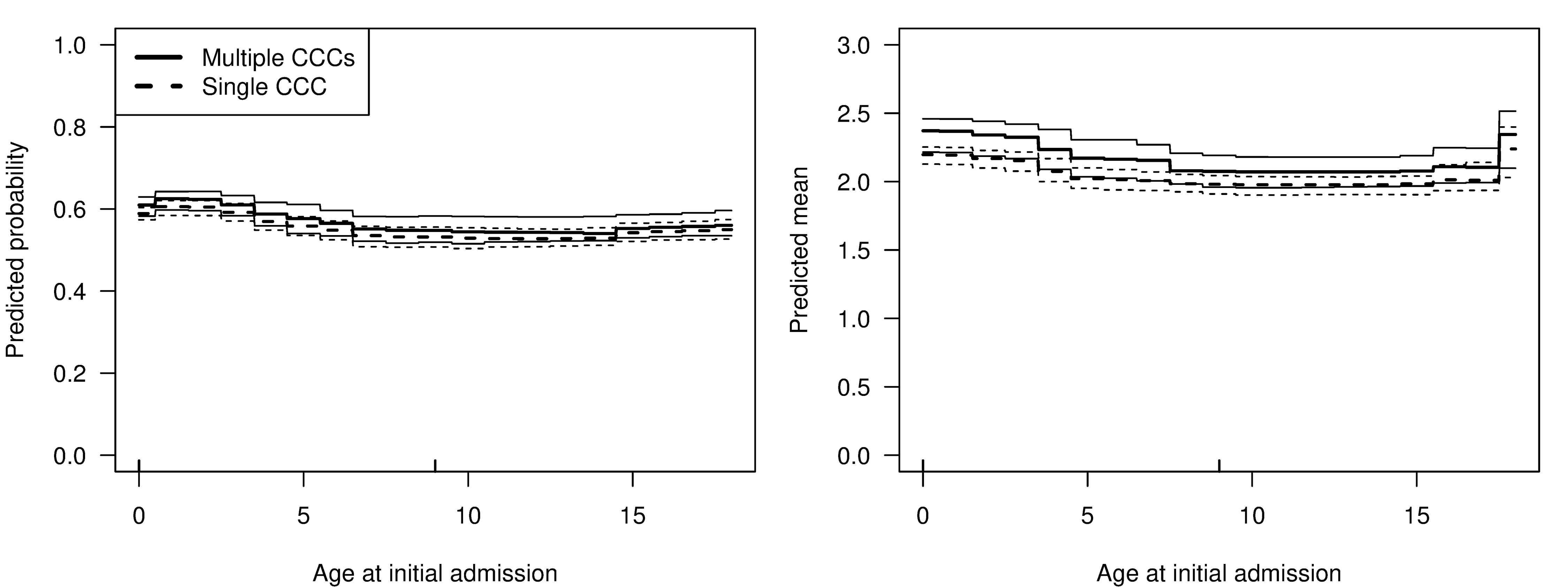

Supplement: S1 Fig — Marginal associations (and 95% confidence intervals) for age at initial admission in the binary (left) and conditional (right) submodels for ED visits in the year following initial discharge. (TIF) [file pone.0312195.s003.tif]

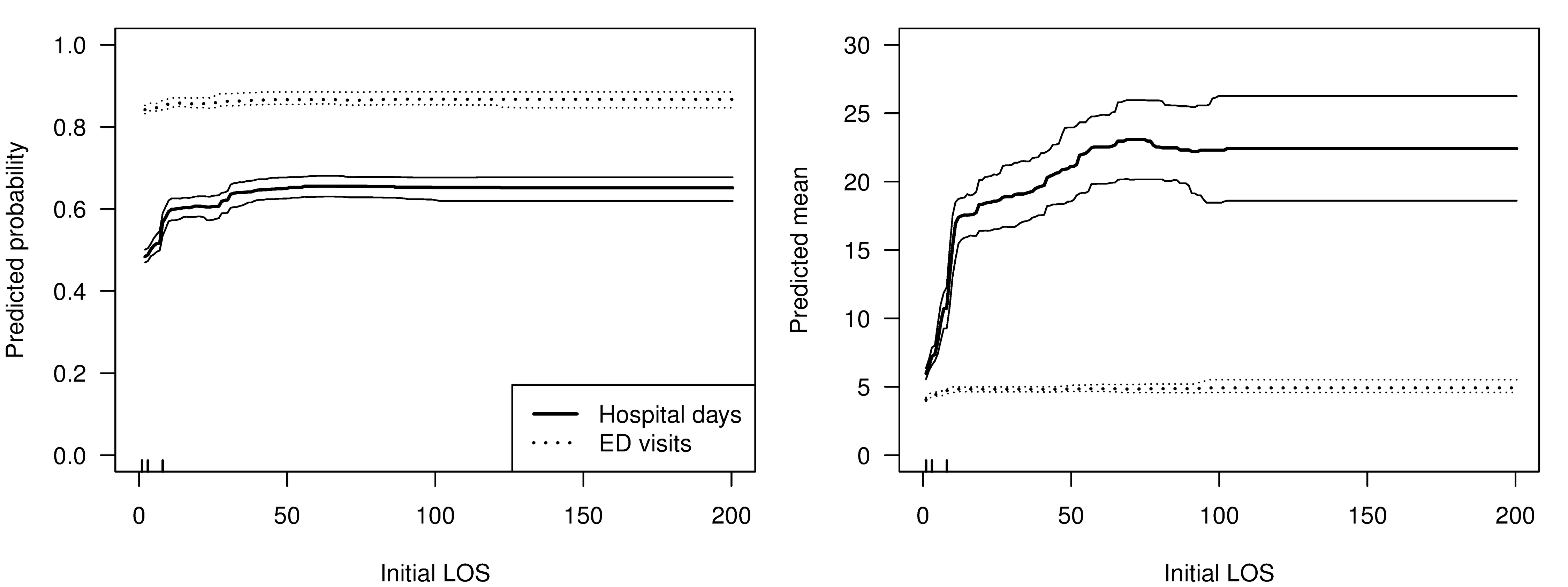

Supplement: S2 Fig — Marginal associations (and 95% confidence intervals) for initial LOS in the binary (left) and conditional (right) submodels in the fifth year following initial discharge. (TIF) [file pone.0312195.s004.tif]

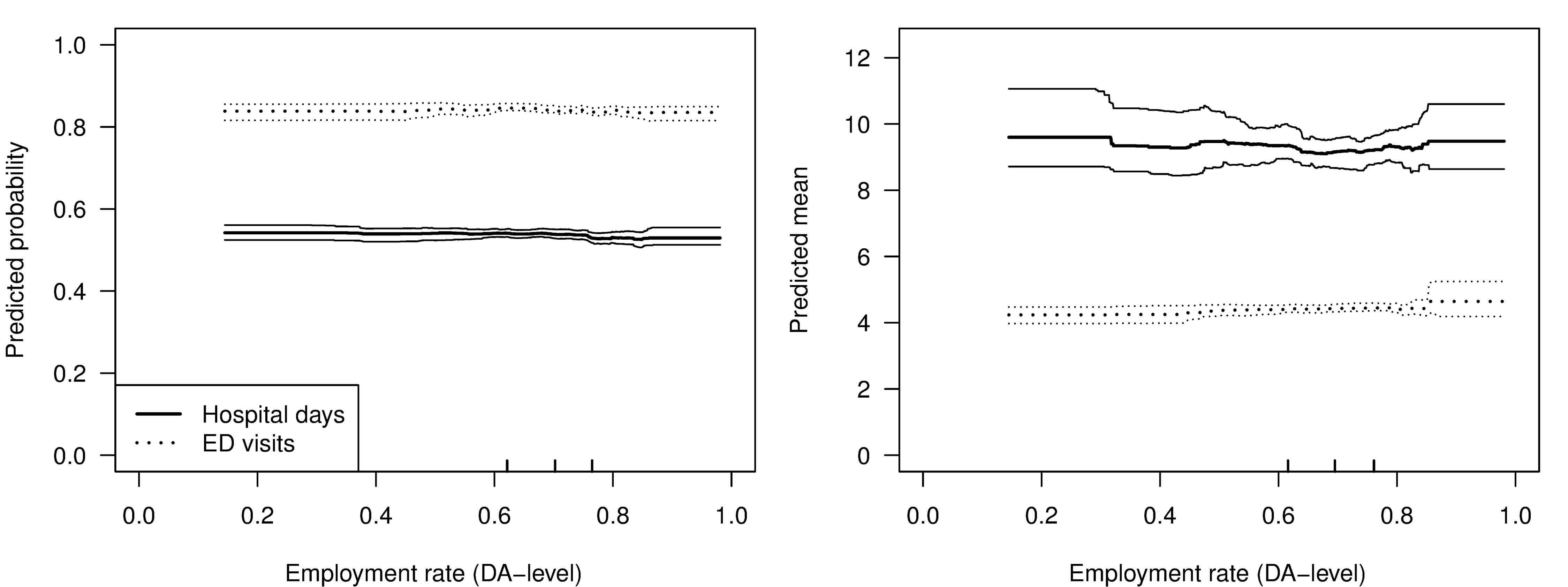

Supplement: S3 Fig — Marginal associations (and 95% confidence intervals) for employment rate (measured at the DA level) in the binary (left) and conditional (right) submodels in the fifth year following initial discharge. (TIF) [file pone.0312195.s005.tif]

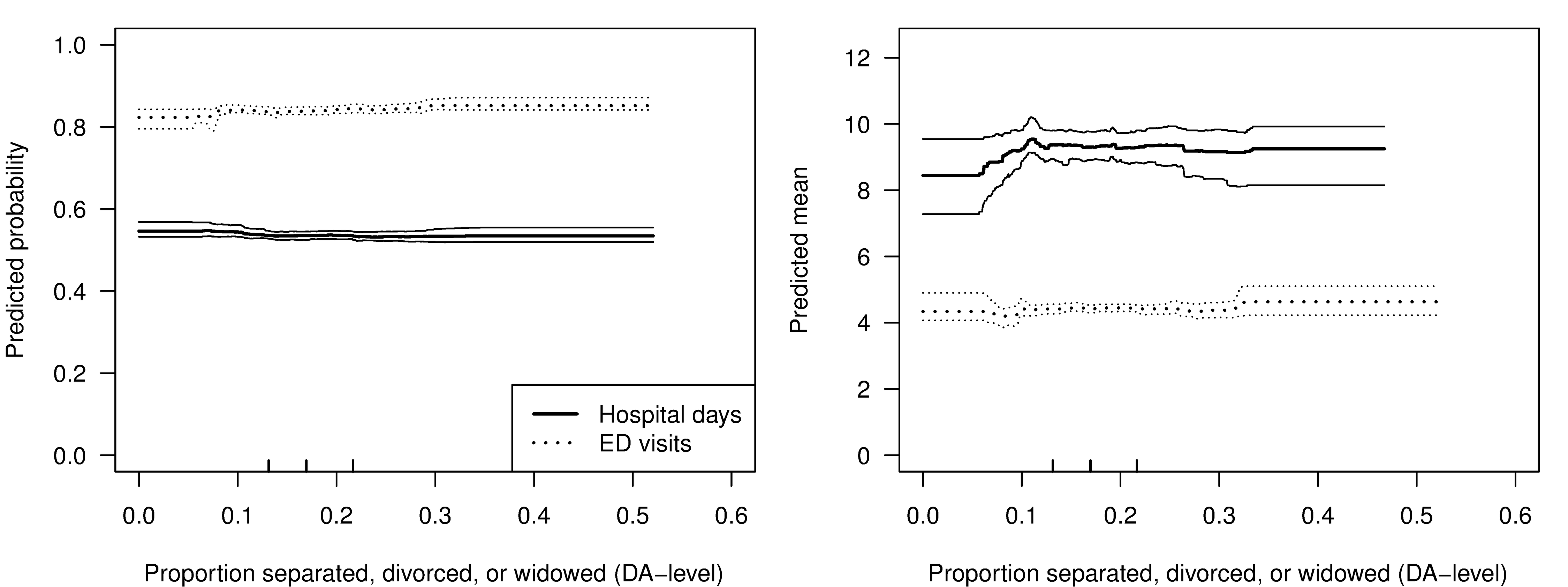

Supplement: S4 Fig — Marginal associations (and 95% confidence intervals) for the proportion of single, divorced, or widowed individuals (measured at the DA level) in the binary (left) and conditional (right) submodels in the fifth year following initial discharge. (TIF) [file pone.0312195.s006.tif]
